# Squash preparation: A reliable diagnostic tool in the intraoperative diagnosis of central nervous system tumors

**DOI:** 10.4103/0970-9371.71870

**Published:** 2010-07

**Authors:** Sumit Mitra, Mohan Kumar, Vivek Sharma, Debasis Mukhopadhyay

**Affiliations:** Department of Pathology, Institute of Medical Sciences, Banaras Hindu University, Varanasi - 221 005, India; 1 Department of Neurosurgery, Institute of Medical Sciences, Banaras Hindu University, Varanasi - 221 005, India; 2 Department of Pathology, Institute of Postgraduate Medical Education and Research, Kolkata - 700 020, India

**Keywords:** Central nervous system tumors, frozen sections, intraoperative diagnosis, squash preparation

## Abstract

**Background:**

Intraoperative cytology is an important diagnostic modality improving on the accuracy of the frozen sections. It has shown to play an important role especially in the intraoperative diagnosis of central nervous system tumors.

**Aim:**

To study the diagnostic accuracy of squash preparation and frozen section (FS) in the intraoperative diagnosis of central nervous system (CNS) tumors.

**Materials and Methods:**

This prospective study of 114 patients with CNS tumors was conducted over a period of 18 months (September 2004 to February 2006). The cytological preparations were stained by the quick Papanicolaou method. The squash interpretation and FS diagnosis were later compared with the paraffin section diagnosis.

**Results:**

Of the 114 patients, cytological diagnosis was offered in 96 cases. Eighteen nonneoplastic or noncontributory cases were excluded. Using hematoxylin and eosin-stained histopathology sections as the gold standard, the diagnostic accuracy of cytology was 88.5% (85/96) and the accuracy on FS diagnosis was 90.6% (87/96). Among these cases, gliomas formed the largest category of tumors (55.2%). The cytological accuracy in this group was 84.9% (45/53) and the comparative FS figure was 86.8% (46/53). In cases where the smear and the FS diagnosis did not match, the latter opinion was offered.

**Conclusions:**

Squash preparation is a reliable, rapid and easy method and can be used as a complement to FS in the intraoperative diagnosis of CNS tumors.

## Introduction

The role of intraoperative pathological diagnosis is crucial in neurosurgery. Besides rapid decision making during neurosurgical procedures, it is also to be ensured that minimum injury is caused to the normal brain structures surrounding the intracranial neoplasm. The role of squash preparations has increased along with the development of stereotactic biopsies, where there has been a significant limitation in the amount of tissue available for intraoperative diagnosis.[1,2] Hence, it has become necessary for pathologists to train themselves in the interpretation of cytomorphological features of various central nervous system (CNS) lesions. The purpose of this study is to assess the diagnostic accuracy of the squash preparation and compare it with that of the frozen section (FS).

## Materials and Methods

This study of 114 patients of CNS space-occupying lesions was conducted over a period of 18 months (September 2004 to February 2006) in our department.

All the cases were open biopsies, and in all 114 cases, squash and FS diagnoses were attempted and compared later with the paraffin section diagnosis. A tiny portion (1–2 mm^3^) of tissue was squashed between two slides to prepare smears as described by Adams *et al*.[3] One smear was immediately immersed in methanol acetone fixative for the “three minute” quick Papanicolaou staining procedure as described by Mouriquand *et al*.[4] The other slide was immersed in 95% ethanol for the conventional Papanicolaou stain in order to compare the former stain with the latter.

A part of the residual tissue was submitted for FS. Five-micrometer sections were cut using the Sakura- coldtome0 and sections were stained by the rapid hematoxylin and eosin (H and E) method. The remaining sample was made into routine paraffin sections and stained by the H and E method. Care was taken to ensure that samples that were smeared, frozen sectioned and paraffin processed were taken from the closest possible areas.

The intraoperative cytological diagnosis offered was based on the quick Papanicolaou staining procedure. In both squash preparations and FS, the diagnosis was considered to be correct if the histology and grading were properly assessed. In all cases, relevant clinical data and radiological findings were available.

## Results

The final histopathology diagnosis is presented in Table 1. In all 114 cases, cytological and FS diagnoses were attempted and compared with paraffin sections. In four patients, no “tumor” was found. Among them, two had tuberculoma cerebellum and two had abscesses in both cerebral hemispheres. In 98 cases, smears were adequate. In two other cases, both squash preparations and FS yielded only necrotic material, with few cells that were diagnosed as glioblastoma multiforme in subsequent paraffin sections. Thus, cytological diagnosis could only be offered in 96 cases.

Diagnostic accuracy on cytology was found to be 88.5% (85/96) and the corresponding diagnostic accuracy on FS was 90.6% (87/96). Of the 96 cases, 53 were gliomas, forming the largest category of tumors (55.2%). The cytological accuracy in this group was 84.9% (45/53) and the comparative FS figure was 86.8% (46/53). The lineage of the gliomas was correctly established in 90.6% (48/53) cases and the grade established in 92.5% (49/53) cases. Among four cases of incorrectly graded gliomas, all were cases of undergrading.

**Table 1:** Spectrum of CNS tumors

Tumor	No.	Percentage
Diffuse astrocytoma WHO grade II	12	10.9
Anaplastic astrocytoma	9	8.2
Glioblastoma multiforme	23	20.9
Pilocytic astrocytoma	5	4.5
Oligodendroglioma	3	2.7
Ependymoma	2	1.8
Meningioma	22	19.9
Schwannoma	13	11.8
Metastatic adenocarcinoma	4	3.6
Medulloblastoma	4	3.6
Hemangioblastoma	3	2.7
Craniopharyngioma	1	0.9
Hemangiopericytoma	1	0.9
Pituitary adenoma	2	1.8
Anaplastic oligoastrocytoma	1	0.9
Adenoid cystic carcinoma	1	0.9
Neurofibroma	2	1.8
Epidermoid cyst	2	1.8

**Figure 1:**
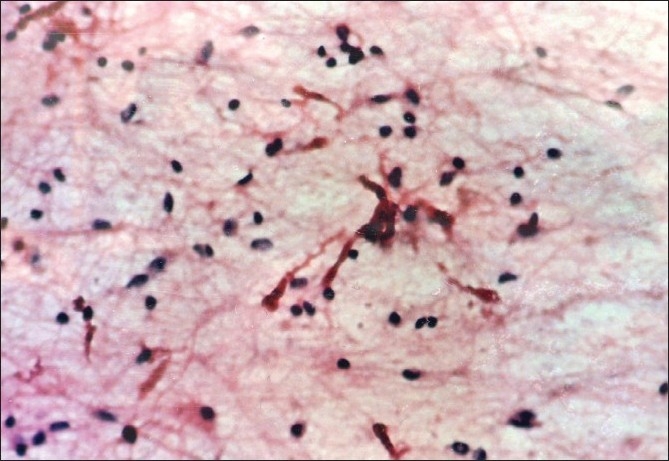
Pilocytic astrocytoma: Squash preparation showing Rosenthal fibres, bipolar astrocytes and thin cytoplasmic fibrillary processes(Pap, ×200)

**Figure 2:**
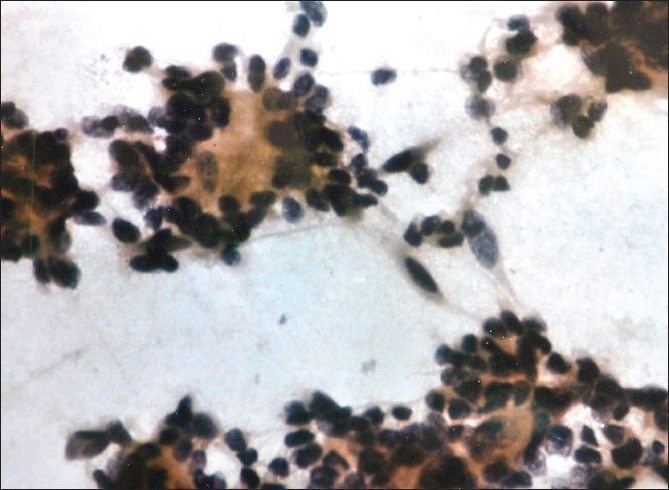
Ependymoma: Squash preparation showing round to oval tumor cells forming rosettes (Pap, ×400)

In squash preparations stained with the quick Papanicolaou staining method, the cytoplasmic fibrillary processes appeared paler and occasionally indistinct compared with the ones stained with the conventional Papanicolaou stain. Other than this, the two staining methods gave similar results. Some morphological aspects of examined smears are depicted in Figures 1 and 2.

Smears were inadequate for 12 tumors (five schwannomas, four meningiomas and one each for neurofibroma, craniopharyngioma and hemangiopericytoma). These tumors were too fibrous to squash. In this group, FS yielded a correct diagnosis in all except two cases. One tumor was interpreted as meningioma in FS, but proved to be hemangiopericytoma in paraffin sections. In the other case, a craniopharyngioma, FS could not be performed. In two other cases, both smears and FS yielded only necrotic material with few cells, which were subsequently diagnosed as glioblastoma in paraffin sections.

The radiological diagnosis correlated well with the final histopathological diagnosis in cases like meningioma, pituitary adenoma, craniopharyngioma and some cases of glioblastoma. However, in most cases of cerebral hemispheric tumors, the findings were nonspecific and differential diagnoses were offered. However, the radiological differential diagnoses included the diagnosis finalized on histopathological evaluation.

## Discussion

The squash technique was introduced in intraoperative neurosurgical diagnosis as early as the ‘30s.[5] However, the advent of stereotactic neurosurgical techniques that produce very small specimens that are difficult to section on the cryostat has resulted in increased popularity of the squash preparation in rapid diagnosis.[2,5] Hence, in order to determine and compare the accuracy of squash and FS diagnoses, we decided to analyze our data collected over a period of 18 months.

In our study, the diagnostic accuracy of the smears was found to be 88.5% and the corresponding diagnostic accuracy on frozen sections was 90.6%. In previously reported studies, the cytomorphological accuracy of diagnosis has varied from 80% to 95%.[1,2,6–12] The diagnoses based on FS are commonly thought to be more accurate than squash preparations, and the reported accuracy varies from 90% to 99%.[2,9,13,14]

The accuracy of cytological diagnosis depends on the consistency of the tissue.[2,5] Soft, friable tissues can be easily made into smears, yielding a good cellularity. The majority of gliomas belong to this category, as do pituitary adenomas, medulloblastomas and metastatic carcinomas. These lesions posed few diagnostic problems as the cell yield was good. On the other hand, in case of firm tissue, such as meningioma and schwannoma, smears were inadequate in 4/22 and 5/13 cases, respectively. Only those tumors that were not very tough could be made into smears in order to allow recognition.

Tissue specific diagnostic accuracy is depicted in Tables 2–4. In smears preparations, the most commonly misdiagnosed entity was the glioblastoma. Two cases were misdiagnosed as metastatic carcinoma. Small cell glioblastomas without fibrillary astrocytic processes was the reason for misdiagnosis in these cases. However, these cases were correctly diagnosed in FS, which showed palisading necrosis and vascular proliferation. Two other cases were underdiagnosed as anaplastic astrocytoma in smears. Both these cases were also misdiagnosed as anaplastic astrocytoma in FS. This discrepancy was probably due to sampling error.

**Table 2:** Cases misdiagnosed on cytology but correctly interpreted on frozen section

Cytology	Frozen section	Paraffin section diagnosis
Diffuse astrocytoma WHO grade II	Anaplastic astrocytoma	Anaplastic astrocytoma
Metastatic adenocarcinoma (2 cases)	Glioblastoma	Glioblastoma
Glioblastoma	Metastatic adenocarcinoma	Metastatic adenocarcinoma
Oligoastrocytoma WHO grade II	Diffuse astrocytoma WHO grade II	Diffuse astrocytoma WHO grade II
Schwannoma	Meningioma	Fibroblastic meningioma

**Table 3:** Cases misdiagnosed on frozen section but correctly interpreted in cytology

Cytology	Frozen section	Paraffin section diagnosis
Oligodendroglioma	Diffuse astrocytoma WHO grade II	Oligodendroglioma
Ependymoma	Metastatic adenocarcinoma	Papillary ependymoma
Diffuse astrocytoma WHO grade II	Anaplastic astrocytoma	Diffuse astrocytoma WHO grade II
Hemangioblastoma	Pilocytic astrocytoma	Hemangioblastoma

**Table 4:** Cases misdiagnosed on both frozen section and cytology

Cytology	Frozen section	Paraffin section diagnosis
Anaplastic astrocytoma (2 cases)	Anaplastic astrocytoma (2 cases)	Glioblastoma
Diffuse astrocytoma WHO grade II	Diffuse astrocytoma WHO grade II	Oligodendroglioma
Diffuse astrocytoma WHO grade II	Diffuse astrocytoma WHO grade II	Anaplastic oligoastrocytoma
Schwannoma	Schwannoma	Fibroblastic meningioma

Grade was again incorrectly assessed in two other cases of anaplastic astrocytoma and anaplastic oligoastrocytoma, both of which were misdiagnosed as diffuse astrocytoma WHO grade II. While the former was correctly diagnosed in FS, in case of the latter, FS interpreted it wrongly as diffuse astrocytoma WHO grade II, probably due to a fault in tissue piece selection.

Incorrect assessment was observed in one case each of oligodendroglioma and diffuse astrocytoma WHO grade II, which were diagnosed as diffuse astrocytoma WHO grade II and oligoastrocytoma WHO grade II, respectively. In case of the former, the error was probably due to the lack of uniformly appearing nuclei and seemingly apparent cytoplasmic processes; in the latter, in many areas of the smear, the cells occurred in sheets and had a rather uniform nuclear appearance. This case of oligodendroglioma was also misdiagnosed in FS as diffuse astrocytoma WHO grade II due to the seemingly mild nuclear pleomorphism. However, the case of diffuse astrocytoma WHO grade II was correctly interpreted in FS.

Two cases of fibroblastic meningioma were misdiagnosed as schwannoma in squash preparations due to the presence of elongated spindly cells and lack of cellular whorls in smears. One of these cases was correctly diagnosed in FS, where cellular whorls were distinct. But, in the other case, even FS diagnosis was erroneous.

Finally, a case of metastatic adenocarcinoma was misdiagnosed in cytology as glioblastoma because of the presence of bizarre type of cells in a necrotic background. This case was correctly interpreted on FS, which displayed the tissue architecture well.

The discrepancies observed between the smear and the permanent preparations are consistent with the nature of errors reported in previous studies, where the reasons usually cited by authors were sampling error, under grading of gliomas and error in recognition of histologic cell type.[1,5,10] That astrocytomas may vary significantly in grade from one area to another within a single tumor is well known.[15,16]

In case of FS, two cases of glioblastoma were misdiagnosed as anaplastic astrocytoma, the error being probably due to improper sampling of tissue. Besides the case of oligodendroglioma discussed earlier, another case of the same was diagnosed as diffuse astrocytoma WHO grade II. The error might be due to the distortion and exaggerated anisocytosis observed in FS.[17] Similarly, a case of diffuse astrocytoma WHO grade II was misinterpreted as anaplastic astrocytoma. This was perhaps due to artefactual atypia in the FS. In cases where the smear and FS diagnosis did not match, the latter opinion was offered in this study. However, in view of the discrepant cases [Table 3], including a case of papillary ependymoma that was misinterpreted as metastatic adenocarcinoma in FS but correctly identified on smear preparation based on true rosettes and benign appearance of cells, each case must be assessed independently.

One case of haemangioblastoma, where the biopsy was probably a peripheral one, showed Rosenthal fibres along with numerous small blood vessels. This case was misdiagnosed as pilocytic astrocytoma in FS; however, squash preparations showed trabeculae of elongated cells amidst densely vascular tissue, cells with round to oval nuclei and ill-defined cytoplasm along with hemosiderin-containing phagocytes thus helping to reach the correct diagnosis.

Problems in FS diagnosis have been faced by other authors too who have reported that although FSs preserved tissue architecture and displayed degree of cellularity, in many cases, especially gliomas, they showed marked freezing artifact and distortion.[15,17]

As the major aim was surgical decompression, the surgeon(s) attempted to remove as much tissue as possible in all the cases in this study. To the best of the authors’ knowledge, there was no negative impact on the type or extent of surgery performed in any of the discrepant cases.

## Conclusions

Intraoperative squash preparations in neurosurgery are not only easy to obtain and inexpensive but also are fairly accurate and dependable tools to diagnose CNS tumors.[1,2,6–11,18] Hence, these can be used for rapid diagnosis in developing countries where supply of electricity is irregular and cost of the cryostat is prohibitive.[19] Also, with the increase in the use of stereotactic biopsy procedures, intraoperative squash preparations will gain further popularity.
